# Lymphopaenia in cardiac arrest patients

**DOI:** 10.1186/s13613-017-0308-z

**Published:** 2017-08-14

**Authors:** Paola Villois, David Grimaldi, Savino Spadaro, Claudia Righy Shinotsuka, Vito Fontana, Sabino Scolletta, Federico Franchi, Jean-Louis Vincent, Jacques Creteur, Fabio Silvio Taccone

**Affiliations:** 1 Department of Intensive Care, Erasme Hospital, Université Libre de Bruxelles (ULB), Route de Lennik, 808, 1070 Brussels, Belgium; 2 Department of Morphological Surgery and Experimental Medicine, Arcispedale Sant’Anna, Università di Ferrara, Via AldoMoro, 8, 44121 Ferrara, Italy; 3 Department of Anesthesia and Intensive Care, Policlinico Santa Maria alle Scotte, Universitá di Siena, Viale Bracci, 14, 53100 Siena, Italy

**Keywords:** Lymphopaenia, Cardiac arrest, Outcome, Prognosis

## Abstract

**Background:**

A decrease in circulating lymphocytes has been described as a marker of poor prognosis after septic shock; however, scarce data are available after cardiac arrest (CA). The aim of this study was to evaluate the impact of lymphopaenia after successful cardiopulmonary resuscitation.

**Methods:**

This is a retrospective analysis of an institutional database including all adult CA patients admitted to the intensive care unit (ICU) between January 2007 and December 2014 who survived for at least 24 h. Demographic, CA-related data and ICU mortality were recorded as was lymphocyte count on admission and for the first 48 h. A cerebral performance category score of 3–5 at 3 months was considered as an unfavourable neurological outcome.

**Results:**

Data from 377 patients were analysed (median age: 62 [IQRs: 52–75] years). Median time to return of spontaneous circulation (ROSC) was 15 [8–25] min and 232 (62%) had a non-shockable initial rhythm. ICU mortality was 58% (*n* = 217) and 246 (65%) patients had an unfavourable outcome at 3 months. The median lymphocyte count on admission was 1208 [700–2350]/mm^3^ and 151 (40%) patients had lymphopaenia (lymphocyte count <1000/mm^3^). Predictors of lymphopaenia on admission were older age, a shorter time to ROSC, prior use of corticosteroid therapy and high C-reactive protein levels on admission. ICU non-survivors had lower lymphocyte counts on admission than survivors (1100 [613–2317] vs. 1316 [891–2395]/mm^3^; *p* = 0.05) as did patients with unfavourable compared to those with favourable neurological outcomes (1100 [600–2013] vs. 1350 [919–2614]/mm^3^; *p* = 0.003). However, lymphopaenia on admission was not an independent predictor of poor outcomes in the entire population, but only among OHCA patients.

**Conclusions:**

A low lymphocyte count is common in CA survivors and is associated with poor outcome after OHCA.

**Electronic supplementary material:**

The online version of this article (doi:10.1186/s13613-017-0308-z) contains supplementary material, which is available to authorized users.

## Background

Despite the high mortality rates still associated with sudden cardiac arrest (CA), advances have been made in recent years, in particular to improve the proportion of patients achieving return of spontaneous circulation (ROSC) after cardiopulmonary resuscitation (CPR) [1]. Although the implementation of high-quality CPR and early defibrillation has increased the number of CA patients being admitted to the hospital, there has been no associated increase in survival rates and neurological recovery among CA survivors [2, 3]. The high mortality rate observed in this patient population is related to the consequences of the global ischaemia–reperfusion process, the so-called post-cardiac arrest syndrome (PCAS), which is characterised by a systemic inflammatory response that may be involved in the development of myocardial dysfunction, brain injury and multiple organ failure [3, 4].

The PCAS is a complex pathophysiological process, which encompasses a generalised activation of immunological and coagulation pathways and has many features common with sepsis [4]. Adrie et al. [4] reported a significant increase in the concentrations of various serum cytokines in CA patients in the first hours after the initial injury, which was more significant in non-survivors than in survivors. Other studies have also shown impaired microcirculatory perfusion and increased plasma endotoxin levels in CA patients, similar to findings in sepsis [5, 6]. Interestingly, sepsis is rapidly accompanied by a state of relative immunosuppression, characterised by a reduction in the number of functional immune cells and T-cell dysfunction [7, 8], which predicts a poor outcome [9].

Among the different components of the immune response, lymphocytes are essential in the organism’s defence against external aggression, by interaction (T cells) with antigen-presenting cells and subsequent activation of the immune response (B and T cells), in a complex interplay of cell-to-cell interactions, production of adhesion molecules and modulation of growth factors [10]. Severe lymphopaenia has been described in almost 30% of patients admitted to the intensive care unit (ICU) with severe sepsis or septic shock and was associated with high plasma levels of tumour necrosis factor-α, interleukin (IL)-6 and IL-10 [11]. Lymphopaenia during sepsis was also associated with features of immunosuppression, such as spontaneous hypothermia and an increased risk of hospital-acquired infections, and was an independent predictor of poor outcome [9, 12]. Persistent lymphopaenia has also been associated with poor outcomes in trauma patients, in particular when an infection occurred during the hospital stay [13]. Although survivors from CA are at high risk of secondary infections, in particular early-onset pneumonia [14], no data are available on the relationship between lymphocyte count and the risk of infection or patient outcome. Thus, the aim of our study was to evaluate the prevalence of lymphopaenia among CA survivors and its association with ICU survival and long-term neurological outcome. We hypothesised that lymphopaenia could be a significant predictor of poor outcome in this setting.

## Methods

### Study population

This study was performed in the Department of Intensive Care at Erasme Hospital. All comatose patients (Glasgow Coma Scale [GCS] score <9) admitted after in-hospital (IHCA) or out-of-hospital (OHCA) CA and surviving for at least 24 h after the arrest were included in an institutional database (January 2007–December 2014) and considered as eligible for the study. Exclusion criteria were missing data for blood count or lymphocyte count on admission.

### Post-resuscitation care

All comatose CA patients were treated with targeted temperature management (TTM; target body temperature 32–34 °C) for 24 h, according to a standardised institutional protocol. Cooling was started immediately after hospital admission using a bolus of cold fluid (in general saline solutions, given as a dose of 20–30 mL/kg over 30 min) and a water-circulating blanket device (Medi-Therm II, Gaymar, USA, or Arctic Sun, Bard, France). Sedation and analgesia consisted of midazolam and morphine, which were adjusted to obtain deep sedation (e.g. severe depression of consciousness during which patients cannot be aroused with repeated or painful stimulation); cisatracurium was administered to control shivering in the induction phase (bolus of 0.15 mg/kg) and then given, if needed, by continuous infusion (1–3 mcg/kg/min). Rewarming (<0.5 °C/h) was achieved passively, and sedation/analgesia was discontinued at normothermia (>37 °C).

Patients were kept in a 30° semi-recumbent position; ventilation was set to target PaCO_2_ between 35 and 45 mmHg and SpO_2_ >94%. Blood glucose was kept between 110 and 150 mg/dL using a local protocol for continuous insulin infusion; enteral nutrition was allowed. Mean arterial pressure was maintained at least above 65 mmHg using volume resuscitation, noradrenaline and/or dobutamine, when needed. Intra-aortic balloon counterpulsation (IABP) or extracorporeal membrane oxygenation (ECMO) was also used in severe cardiogenic shock.

### Data collection

We collected data on demographics, pre-existing chronic diseases and immunosuppressive treatment, and CPR (initial rhythm, bystander CPR, time to ROSC, total adrenaline dose) in all patients. A complete blood count, including neutrophil and lymphocyte cell counts, and C-reactive protein (CRP) concentration were recorded on ICU admission and during the first 48 h. We also recorded the use of any other immunosuppressive drugs, vasopressors, mechanical ventilation and continuous renal replacement therapy (CRRT) during the ICU stay. Lactate concentrations were also collected on admission. The development of infections during the ICU stay was recorded; survival was recorded at ICU discharge and ICU length of stay was noted. Neurological evaluation at 3 months after CA was assessed using the cerebral performance category score (CPC; 1  =  no or mild neurological disability, 2  =  moderate neurological disability, 3  =  severe neurological impairment, 4  =  vegetative state, 5  =  death). The CPC evaluation was assessed during follow-up visits or by telephone interview with the general practitioner.

### Definitions

Lymphopaenia was defined as an absolute lymphocyte count of <1000/mm^3^ cells; severe lymphopaenia was defined as an absolute lymphocyte count of <500/mm^3^ cells [15]. Patients were subsequently divided into four groups according to the lymphocyte levels during the study period: “group 1” included patients with persistent lymphopaenia throughout the 48 h; “group 2” included patients with lymphopaenia on admission but with normal lymphocyte counts at 48 h; “group 3” included those who had normal lymphocyte counts throughout the 48-h study period; and “group 4” included those with normal lymphocyte counts on admission but who had developed lymphopaenia by 48 h.

Favourable 3-month neurological outcome was defined as a CPC of 1–2 at 3 months and unfavourable neurological outcome as a CPC of 3–5. The diagnosis of infection was made according to the CDC/NHSN criteria [16]. Acute kidney injury (AKI) was defined as a daily urine output <0.5 mL/kg/h and/or an increase in serum creatinine level by at least 0.3 mg/dL or >1.5 times increase from baseline values, as previously reported [17]. Shock was defined as the need for vasopressor agents for more than 6 h.

### Statistical analysis

Statistical analyses were performed using the SPSS 24.0 for Windows NT software package (SPSS Inc, Chicago, IL, USA). Descriptive statistics were computed for all study variables. The Kolmogorov–Smirnov test was used and histograms and normal-quantile plots were examined to verify the normality of distribution of continuous variables. Discrete variables were expressed as counts (percentage) and continuous variables as means  ±  SD or median (25th to 75th percentiles). Demographics and clinical differences between groups were assessed using the Chi-square test, Fisher’s exact test, Student’s *t* test or Mann–Whitney *U* test, as appropriate. The significance of differences among the four groups was analysed using two-way (time and group) analysis of variance for repeated measures (ANOVA), followed by Bonferroni post hoc analysis. Multivariable logistic regression analysis with lymphopaenia on admission as the dependent variable was performed in all patients; co-linearity between variables was excluded prior to modelling; only variables associated with a higher risk of ICU mortality (*p* < 0.2) on a univariate basis were introduced in the multivariable model. Odds ratios (OR) with 95% confidence intervals (CI) were computed. The same analysis was then performed to identify independent predictors of survival and neurological outcome and for all measured outcome considering only IHCA or OHCA patients. Also, an additional analysis on the independent predictors of lymphopaenia on admission with the exclusion of patients under corticosteroids or other immunosuppressive therapies was performed. A *p* value <0.05 was considered as statistically significant.

## Results

Of the 424 eligible patients admitted during the study period, 23 died within the first 24 h and 24 were excluded for missing data, so that 377 were included in the final analysis. The main characteristics of the study population (age 62 [52–72] years—OHCA 56%) are given in Table 1. Median time to ROSC was 15 min and 232 (62%) patients had a non-shockable initial rhythm; 150 (40%) patients had a non-cardiac origin of arrest. ICU mortality was 58% (*n* = 217) and 131 patients (38%) had a favourable neurological outcome.

**Table 1:** Characteristics of study population according to the presence of lymphopaenia on admission

	All (*n* = 377)	Lymphopaenia (*n* = 151)	No lymphopaenia (*n* = 226)	*p* value
Age (years)	62 [52–75]	69 [54–78]	59 [51–71]	<0.001
Weight (kg)	76 [67–85]	75 [67–82]	77 [67–88]	0.22
Male, *n* (%)	263 (70)	105 (70)	158 (70)	0.51
Witnessed CA, *n* (%)	318 (84)	132 (87)	186 (82)	0.12
Bystander CPR, *n* (%)	249 (66)	110 (73)	139 (62)	0.02
Time to ROSC (min)	15 [8–25]	10 [5–21]	18 [10–27]	<0.001
Adrenaline (mg)	3 [2–6]	3 [1–5]	4 [2–7]	<0.001
Out-of-hospital CA, *n* (%)	211 (56)	60 (40)	151 (67)	<0.001
Cardiac origin of arrest, *n* (%)	227 (60)	80 (53)	147 (65)	0.01
Shockable rhythm, *n* (%)	145 (38)	41 (27)	104 (46)	<0.001
Comorbidities				
Chronic heart failure, *n* (%)	81 (21)	37 (25)	44 (19)	0.15
Hypertension, *n* (%)	171 (45)	78 (52)	93 (41)	0.03
Coronary artery disease, *n* (%)	164 (44)	62 (41)	102 (45)	0.25
Diabetes, *n* (%)	92 (24)	48 (32)	44 (19)	0.01
COPD/asthma, *n* (%)	66 (18)	31 (21)	35 (15)	0.13
Neurological disease, *n* (%)	64 (17)	31 (21)	32 (14)	0.05
Chronic renal disease, *n* (%)	67 (18)	36 (24)	31 (14)	0.008
Liver cirrhosis, *n* (%)	22 (6)	14 (9)	8 (4)	0.02
HIV, *n* (%)	1 (0)	1 (1)	0	0.40
Corticosteroid therapy, *n* (%)	73 (19)	41 (27)	32 (14)	0.001
Other immunosuppressive agents, *n* (%)	15 (4)	11 (7)	4 (2)	0.008
Initial blood analysis				
White blood cells count (/mm^3^)	12,200 [9000–16,700]	10,700 [7700–14,500]	13,100 [9925–17,475]	0.06
Neutrophils (/mm^3^)	9400 [6300–13,500]	9100 [6500–13,200]	9915 [5947–13,700]	<0.001
Haemoglobin (g/dL)	12.0 [9.9–13.7]	10.6 [8.9–12.9]	12.8 [10.8–14.1]	0.04
Platelets (*10^3^/mm^3^)	193 [127–264]	158 [100–230]	210 [147–285]	<0.001
C-reactive protein (mg/dL)	9 [2–52]	29 [4–93]	5 [2–22]	<0.001
Lactate (mEq/L)	4.3 [2.8–7.9]	4.1 [2.6–7.8]	4.5 [2.9–7.9]	0.20
During ICU stay				
Infection, *n* (%)	215 (57)	100 (66)	115 (51)	0.002
IABP, *n* (%)	27 (7)	6 (4)	21 (9)	0.04
ECMO, *n* (%)	51 (14)	23 (15)	28 (12)	0.26
Shock, *n* (%)	205 (54)	90 (60)	115 (51)	0.06
Acute kidney injury, *n* (%)	228 (60)	100 (66)	128 (57)	0.04
CRRT, *n* (%)	57 (15)	30 (20)	27 (12)	0.03
Vasopressor therapy, *n* (%)	274 (73)	120 (79)	154 (68)	0.01
Dobutamine therapy, n (%)	200 (53)	85 (56)	115 (51)	0.18
Outcomes				
Total ICU stay (days)	4 [2–8]	4 [2–8]	4 [2–8]	0.93
ICU mortality, *n* (%)	217 (58)	95 (63)	122 (54)	0.05
Hospital mortality, *n* (%)	235 (62)	103 (68)	132 (58)	0.03
Favourable outcome at 3 months, *n* (%)	131 (35)	43 (28)	88 (39)	0.02

*CA* cardiac arrest, *CPR* cardiopulmonary resuscitation, *ROSC* return of spontaneous circulation, *COPD* chronic obstructive pulmonary disease, *IABP* intra-aortic balloon pump counterpulsation, *ECMO* extracorporeal membrane oxygenation, *CRRT* continuous renal replacement therapy, *ICU* intensive care unit, *HIV* human immunodeficiency virus

### Lymphocyte count

**Fig. 1:**
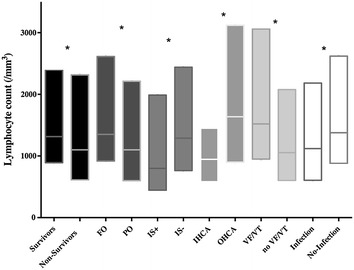
Differences in lymphocyte counts on admission among survivors versus non-survivors; patients with favourable (FO) versus unfavourable neurological outcome (UO); patients receiving immunosuppressive agents (IS+) versus others (IS−); patients with in-hospital (IHCA) versus out-of-hospital cardiac arrest (OHCA); patients with shockable rhythms (VF/VT) versus others (no VF/VT); patients who developed infection versus those without infection. Data are presented as median and 25th (*lower limit*) and 75th percentiles (*upper limit*). *p < 0.05 for lymphocyte count

The median white blood cell count on the day of admission was 12,200 [8700–16,775]/mm^3^, including a lymphocyte count of 1100 [600–2208]/mm^3^. The lymphocyte count was lower in patients previously treated with immunosuppressive agents (*n* = 88) compared to the other patients (800 [445–1989]/mm^3^ vs. 1290 [765–2439]/mm^3^; *p* = 0.004), in patients with IHCA compared to those with OHCA (946 [583–1450]/mm^3^ vs. 1637 [900–3120]/mm^3^; *p* < 0.001) and in patients with non-shockable rhythms compared to those with shockable rhythms (1055 [601–2078]/mm^3^ vs. 1520 [950–3060]/mm^3^; *p* < 0.001—Fig. 1).

**Fig. 2:**
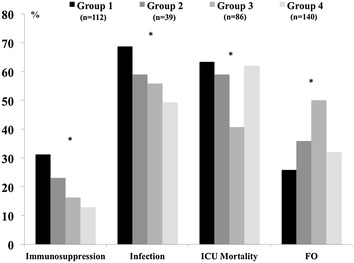
Proportion of patients on immunosuppressive therapy, developing infections, non-survivors and with favourable neurological outcome (FO) according to the different groups of lymphocyte levels over the first 48 h after arrest: “group 1” included patients with persistent lymphopaenia throughout the 48 h; “group 2” included patients with lymphopaenia on admission but with normal lymphocyte counts at 48 h; “group 3” included those who had normal lymphocyte counts throughout the 48-h study period; and “group 4” those with normal lymphocyte counts on admission but who had developed lymphopaenia by 48 h

When grouped according to the change in lymphocyte count during the 48-h period, 112 patients (30%) were in group 1, 39 (25%) in group 2, 86 (23%) in group 3 and 140 (37%) in group 4. The proportions of patients receiving immunosuppressive therapy, of non-survivors, of patients with unfavourable outcome and of patients developing an infection during the ICU stay were significantly higher in group 1 than in the other groups (Fig. 2).

### Lymphopaenia

A total of 151 (40%) patients had lymphopaenia, which was severe in 48 patients. Patients with lymphopaenia on admission were older, had a shorter time to ROSC and were more likely to have had IHCA, a non-cardiac aetiology of CA, bystander CPR and a non-shockable initial rhythm than patients without lymphopaenia (Table 1). Comorbid hypertension, diabetes and immunosuppressive therapy were more common in patients with lymphopaenia than in those without. Patients with lymphopaenia on admission also had a lower neutrophil count and haemoglobin concentration on admission and higher CRP concentrations. Lymphopaenic patients more often developed an infection and AKI during the study period and more frequently required vasopressors and CRRT.

### Lymphopaenia, survival and neurological outcome

ICU non-survivors had a lower lymphocyte count on admission than ICU survivors (1100 [613–2317]/mm^3^ vs. 1316 [891–2395]/mm^3^; *p* = 0.048). The non-survivors were older, more frequently had unwitnessed CA, had a longer time to ROSC and had a higher occurrence of non-cardiac origin of arrest and non-shockable rhythms than survivors (Table 2). Non-survivors had higher lactate levels on admission and more frequently had shock or AKI during the ICU stay than survivors. These patterns were similar in patients with unfavourable compared to those with favourable outcomes (Table 2).

**Table 2:** Characteristics of study population according to ICU survival and long-term neurological outcome

	ICU survivors (*n* = 160)	ICU non-survivors (*n* = 217)	Favourable outcome (*n* = 131)	Unfavourable outcome (*n* = 246)
Age (years)	59 [49–71]	66 [54–78]*	58 [50–70]	66 [53–77]*
Weight (kg)	77 [70–85]	75 [65–85]	78 [70–85]	75 [65–85]
Male, *n* (%)	118 (74)	145 (67)	97 (74)	166 (67)
Witnessed CA, *n* (%)	144 (90)	174 (80)*	118 (90)	200 (80)*
Bystander CPR, *n* (%)	122 (76)	127 (59)*	101 (77)	148 (60)*
Time to ROSC (min)	12 [5–20]	18 [10–25]*	12 [5–20]	17 [10–25]*
Adrenaline (mg)	2 [1–4]	4 [2–7]*	2 [1–4]	4 [2–6]*
Out-of-hospital CA, *n* (%)	86 (54)	125 (57)	74 (56)	137 (56)
Cardiac origin of arrest, *n* (%)	110 (69)	117 (54)*	102 (78)	133 (54)*
Shockable rhythm, *n* (%)	89 (56)	56 (26)*	82 (63)	63 (26)*
Comorbidities				
Chronic heart failure, *n* (%)	33 (21)	48 (22)	27 (21)	54 (22)
Hypertension, *n* (%)	76 (48)	95 (44)	60 (46)	111 (45)
Coronary artery disease, *n* (%)	67 (42)	97 (45)	54 (41)	110 (45)
Diabetes, *n* (%)	35 (22)	57 (26)	25 (19)	67 (27)
COPD/asthma, *n* (%)	26 (16)	40 (18)	19 (15)	47 (19)
Neurological disease, *n* (%)	22 (14)	42 (19)	13 (10)	51 (21)*
Chronic renal disease, *n* (%)	26 (16)	41 (19)	20 (15)	47 (19)
Liver cirrhosis, *n* (%)	7 (4)	15 (7)	4 (3)	18 (7)
HIV, *n* (%)	1 (1)	–	1 (1)	–
Corticosteroid therapy, *n* (%)	25 (16)	48 (22)	20 (15)	53 (22)
Immunosuppressive agents, *n* (%)	5 (3)	10 (5)	3 (2)	12 (5)
Lactate on admission (mEq/L)	3.8 [2.4–5.7]	4.9 [3.2–8.7]*	3.9 [2.5–5.7]	4.6 [3–8.5]*
During ICU stay				
Infection, *n* (%)	105 (66)	110 (51)*	83 (63)	132 (54)
IABP, *n* (%)	9 (6)	18 (8)	7 (5)	20 (8)
ECMO, *n* (%)	19 (12)	32 (15)	18 (14)	33 (13)
Shock, *n* (%)	68 (43)	137 (63)*	58 (44)	147 (60)*
Acute kidney injury, *n* (%)	81 (51)	147 (68)*	65 (50)	163 (66)*
CRRT, *n* (%)	23 (14)	34 (16)	19 (15)	38 (15)
Vasopressor therapy, *n* (%)	99 (62)	175 (81)*	81 (62)	193 (78)*
Dobutamine therapy, *n* (%)	81 (51)	119 (55)	66 (50)	134 (54)
Total ICU stay	6 [3–11]	2 [2–5]*	5 [3–11]	3 [2–5]*

*CA* cardiac arrest, *CPR* cardiopulmonary resuscitation, *ROSC* return of spontaneous circulation, *COPD* chronic obstructive pulmonary disease, *IABP* intra-aortic balloon pump counterpulsation, *ECMO* extracorporeal membrane oxygenation, *CRRT* continuous renal replacement therapy, *ICU* intensive care unit, *HIV* human immunodeficiency virus

* *p* < 0.05 in survivors versus non-survivors OR favourable versus unfavourable outcome

### Multivariable analyses to predict lymphopaenia

In a multivariable logistic regression analysis, older age, a shorter time to ROSC, comorbid use of corticosteroid therapy and high CRP levels on admission were independently associated with the presence of lymphopaenia on admission (Table 3); IHCA was not associated with the occurrence of lymphopenia (OR 1.741 [−3.032 to 49.710]; *p* = 0.083). In IHCA patients, older age, a shorter time to ROSC, high white blood cells count and CRP levels on admission and the use of corticosteroids were independently associated with the presence of lymphopaenia on admission (Additional file 1: Supplemental Tables 1 and 5). In OHCA patients, a shorter time to ROSC, high white blood cells and CRP levels as well as low platelet count on admission were independently associated with the presence of lymphopaenia on admission (Additional file 1: Supplemental Tables 1 and 5). Finally, older age, a shorter time to ROSC, the presence of a non-shockable rhythm, high white blood cells count and CRP levels or low platelets count on admission and the use of corticosteroids were independently associated with the presence of lymphopaenia on admission in those patients without corticosteroids or immunosuppressive therapy (Additional file 1: Supplemental Tables 4 and 8).

**Table 3:** Multivariable regression analysis to identify independent predictors of lymphopaenia on admission

	Lymphopaenia on admission			
	*p* value	OR	95% CI for OR	
			Lower	Upper
Age (years)	0.012	1.023	1.005	1.042
Time to ROSC (min)	0.001	0.960	0.936	0.984
Previous corticosteroid therapy	0.048	2.040	1.007	4.131
CRP (mg/dL)	0.007	1.005	1.002	1.009

Hosmer and Lemeshow goodness-of-fit test Chi-squared = 4.93 (*p* = 0.76). This model has a 69% correct classification (48% for lymphopenia and 83% for non-lymphopenia)

*ROSC* return of spontaneous circulation, *CRP* C-reactive protein

### Multivariable analyses to predict ICU mortality

Using the same statistical approach, older age, the absence of bystander CPR, a non-shockable initial rhythm, a non-cardiac aetiology of the arrest, high blood lactate levels on admission and the use of vasopressors were independent predictors of ICU mortality (Table 4), but lymphopaenia was not (OR 1.367 [0.787–2.567; *p* = 0.26). In IHCA patients, the absence of bystander CPR, a non-shockable rhythm, high epinephrine dose and the development of shock were independently associated with ICU mortality (Additional file 1: Supplemental Tables 2, 6 and 7), but not lymphopaenia (OR 1.319 [0.708–2.458]; *p* = 0.38). In OHCA patients, older age, the absence of bystander CPR, a non-cardiac origin of the arrest, a non-shockable rhythm and high blood lactate levels on admission were independent predictors of ICU mortality (Additional file 1: Supplemental Tables 3, 6 and 7); lymphopaenia was not associated with ICU mortality (OR 1.912 [0.876–3.621]; *p* = 0.09).

**Table 4:** Multivariable regression analysis to identify independent predictors of ICU mortality and unfavourable neurological outcome at 3 months after cardiac arrest

	ICU mortality			
	*p* value	OR	95% CI for OR	
			Lower	Upper
Age (years)	0.001	1.042	1.033	1.055
Bystander CPR	0.006	0.441	0.218	0.755
Non-cardiac aetiology	0.015	2.069	1.345	4.568
Non-shockable rhythm	0.001	2.687	1.876	4.325
Vasopressor use	0.02	2.142	1.356	8.567
Lactate on admission (mEq/L)	0.003	1.113	1.096	1.301

	Unfavourable neurological outcome			
	*p* value	OR	95% CI for OR	
			Lower	Upper
Age (years)	0.001	1.032	1.024	1.057
Bystander CPR	0.008	0.337	0.219	0.785
Non-shockable rhythm	<0.001	2.177	1.765	3.987

ICU mortality: Hosmer and Lemeshow goodness-of-fit test Chi-squared = 5.16 (*p* = 0.23). This model has a 71% correct classification (57% for survivors and 81% for non-survivors)

Neurological outcome: Hosmer and Lemeshow goodness-of-fit test Chi-squared = 9.18 (*p* = 0.33). This model has a 73% correct classification (48% for good neurological outcome and 82% for poor neurological outcome)

*CPR* cardiopulmonary resuscitation

### Multivariable analyses to predict poor long-term neurological outcome

In the multivariable analysis, older age, the absence of bystander CPR and a non-shockable initial rhythm were independent predictors of an unfavourable neurological outcome (Table 4); lymphopaenia was not significantly associated with unfavourable outcome either (OR 1.228 [0.723–3.167; *p* = 0.28). In IHCA patients, the absence of bystander CPR and a non-shockable rhythm were independently associated with UO (Additional file 1: Supplemental Tables 2, 6 and 7), but not lymphopaenia (OR 1.219 [0.719–2.973]; *p* = 0.36). In OHCA patients, older age, a non-cardiac origin of the arrest, lymphopaenia on admission and high epinephrine doses were independent predictors of UO (Additional file 1: Supplemental Tables 3, 6 and 7).

## Discussion

In this study, 40% of post-CA patients had lymphopaenia, and in one-third of them, this persisted through the first 48 h after admission. Predictors of lymphopaenia on admission were older age, a shorter resuscitation time, a history of corticosteroid therapy and high levels of the inflammatory marker (e.g. CRP). Lymphopaenia was more frequent in patients with poor outcome but was not an independent predictor of mortality or unfavourable neurological outcome, unless in the subgroup of OHCA patients.

The occurrence of lymphopaenia and its impact on survivors of CA has not been well studied. In a pig model of prolonged CA and CPR, Gu et al. reported a high degree of splenic lymphocyte apoptosis, which was initiated by activation of the Bcl-2/Bax mitochondrial pathway [18]. These authors also described a reduction in CD4^+^/CD8^+^ T lymphocytes and a shift in these cells from an anti-inflammatory (Th-2) to a pro-inflammatory (T-helper [Th]-1) state in the myocardium [19, 20]. In mice submitted to global cerebral ischaemia secondary to CA within 3 h after resuscitation, Deng et al. [21] showed the presence of infiltrating peripheral CD4^+^ T lymphocytes in the brain. These experimental data indicate that early disturbances of immunological function after ROSC are associated with an increased production of inflammatory mediators and lymphocyte apoptosis, as well as an intense cardiac and neuroimmune response in which infiltrating T cells may play a key role. Few data are available in humans. In 50 OHCA patients, Venet et al. [22] reported a moderate decrease in the number of circulating CD4^+^ T lymphocytes although total lymphocyte count was normal. Nevertheless, the high mortality rate in that study (90%) prevented a comparison of lymphocyte levels in survivors and non-survivors. Indeed, as clinical studies have demonstrated that PCAS is characterised by high plasma cytokine and endotoxin levels, future studies should better characterise how these abnormalities may alter lymphocyte sub-populations and function in CA survivors and whether these changes are reversible or can be influenced by therapeutic interventions [23, 24].

Lymphopaenia has been widely described in patients with sepsis [12, 25, 26]. After an initial predominant pro-inflammatory phase, many septic patients develop persistent immunosuppression, characterised by increased inhibitory receptors on T cells and antigen-presenting cells, decreased production of pro-inflammatory cytokines, expansion of myeloid-derived suppressor cells, and apoptosis-related loss of T and B-lymphocytes and dendritic cells [7, 27]. Induction of lymphocyte apoptosis increased mortality and prevented bacteraemic control in septic animals [28]. The detrimental effects of apoptosis are not only related to the severe loss of immune cells but also the impact that apoptotic cell uptake has on the surviving immune cells. As such, uptake of apoptotic cells by monocytes, macrophages and dendritic cells results in immune tolerance and cellular anergy, which is associated with increased IL-10 production and the induction of a Th-2 cell immune phenotype; the net result of these changes is that the surviving phagocytic cells cannot provide adequate defence against infection [29].

In our study, patients with lymphopaenia were older than those without. Lymphopaenia is a common finding in elderly hospitalised patients and has been associated with poor outcome in these patients [30]. There is a significant interaction between the immune system and the ageing process, which may also influence the occurrence of chronic diseases; in particular, thymic demise represents an important phenomenon that can cause a reduction in T-cell count and peripheral proliferation of pre-existing T-cell clones, which can trigger limited immune reactivity damage-associated molecular patterns [31]. As expected, previous therapy with corticosteroids was also an independent determinant of lymphopaenia [32]. Data on the prevalence of lymphopaenia among corticosteroid users are scarce and biased by the presence of concomitant malignancies, administration of chemotherapy or other immunosuppressive drugs. The mechanisms include increased Th-2 cells with reduction in pro-inflammatory circulating cytokines or externalisation of phosphatidylserine, which may trigger cellular apoptosis, in particular of CD4^+^ cells [33]. Nevertheless, we found an association between increasing CRP levels and the risk of lymphopaenia. This is probably due to increased production of IL-6, which has been shown to be an independent predictor of poor outcome in CA patients [34]. In a recent study, the injection of endotoxin in healthy volunteers was associated with systemic inflammation, which triggered the occurrence of lymphopaenia, by an increase in anti-inflammatory regulatory T cells and a relative functional impairment of T-cell cytokine production, despite detectable levels of plasma pro-inflammatory cytokines [35]. Predictors of lymphopaenia in patients without previous immunosuppressive therapy were similar to those of the entire cohort, underlying again their relevance independently of patients’ immune status.

Interestingly, a shorter time to ROSC was another variable associated with lymphopaenia. We may have expected that the severity of the ischaemic injury, which is related to the longer duration of CPR, would have been associated with more severe apoptosis of T cells and have induced a low lymphocyte count in this setting. In stroke, although ischaemic cerebral damage may lead to suppression of peripheral immune responses, which predisposes to infection, no association was found between the extension of the infarct area and functional immune alterations [36]. The higher prevalence of IHCA in the lymphopaenia group with a shorter intervention time and CPR may also explain this finding. Additional studies should evaluate the complex interplay between pre-existing comorbid conditions and acute cerebral injury and the development of altered immune responses in CA patients.

There was no independent association between lymphopaenia and poor outcome. In patients with sepsis and septic shock, a low lymphocyte count was a strong predictor of poor outcome, even better than increased neutrophil cells [12]. Although sepsis has many common features with the PCAS, lymphocyte count and function may play a marginal prognostic role in CA survivors. One possible explanation could be that the severity of the initial anoxic brain injury (i.e. bystander CPR and time to ROSC) was not correlated with the occurrence of lymphopaenia. Moreover, many patients died within the first 2–3 days after arrest because of significant cardiovascular impairment or extended post-anoxic brain injury [37], limiting the impact of lymphopaenia on the occurrence of secondary infections or delayed organ dysfunction. Nevertheless, we observed that in the subgroup of OHCA patients, lymphopaenia on admission was associated with UO. This suggests that factors as the underlying different causes of the arrest between IHCA or OHCA or the management of CPR according to arrest location (i.e. different response time and quality of CPR) are an important determinant of biological biomarkers of poor prognosis and that routine monitor of the lymphocyte count in OHCA patients could be considered. However, future research should focus on a better characterisation of lymphocytes sub-populations, in relationship with other biomarker of “immunosuppression” (i.e. HLA-DR on monocytes) to better understand the potential prognostic and therapeutic role of lymphopaenia in this patients’ population.

Our study has some limitations. Firstly, it was a retrospective, single-centre study, which may limit the generalisability of our conclusions; this can, however, also be an advantage, as patients were treated according to a local protocol of PCAS management, thus reducing heterogeneity. Secondly, we did not record the exact time of no flow or the quality of CPR, which may influence the inflammatory response after reperfusion. Thirdly, all patients were treated with TTM so that we cannot draw any conclusions on lymphopaenia in normothermic CA patients or on the effects of TTM use, which may blunt the inflammatory response after rewarming, on the impact of lymphopaenia. Forth, causes of CA and lymphopaenia may differ between IHCA and OHCA. Also, it is possible that lymphopaenia may have preceded the arrest in some CA victims, such as in case of IHCA. Although location of arrest was not a significant predictor of lymphopaenia in our study, this might also be due to a lack of power and this question should be further addressed in future larger cohorts. Fifth, some patients, especially if suffering from IHCA, may have sepsis prior to ICU admission, and this could have been a significant trigger to lymphopaenia. Unfortunately, sepsis is widely under-recognised outside the ICU, so that this variable could not be reliably assessed in our database. Finally, we perform a multivariate model to identify variables associated with the occurrence of lymphopaenia, but we did not specifically investigate the lymphocyte count as a continuous variable. Although this might appear more appropriate, clinicians would be more interested in the presence of a “lymphopaenia”, its related risk factors and potential consequences rather than considering the absolute lymphocytes count.

## Conclusions

Lymphopaenia is a common finding in CA survivors, in particular among those with an initial non-shockable rhythm, in-hospital CA and previous immunosuppressive therapy. A lower lymphocyte count on admission was associated with poor outcome, but was not an independent predictor of mortality or neurological recovery. Future studies should better characterise the immune response in patients resuscitated after CA to improve understanding of the pathophysiology of these findings and their potential therapeutic interventions.

## Additional file

**Additional file 1.** This documents includes comparisons between patients with in-hospital (IHCA) and out-of-hospital cardiac arrest (OHCA—Suppl Table 1); analysis of mortality and neurological outcome in IHCA (Suppl Table 2) and OHCA (Suppl Table 3) patients; the characteristics of lymphopenic patients after having excluded those receiving immunosuppressive therapies (Suppl Table 4); a multivariable regression analysis to identify independent predictors of lymphopenia on admission in in-hospital (IHCA) or out-of-hospital (OHCA) cardiac arrest (Suppl Table 5); a multivariable regression analysis to identify independent predictors of ICU outcome in in-hospital (IHCA) or out-of-hospital (OHCA) cardiac arrest (Suppl Table 6); a multivariable regression analysis to identify independent predictors of long-term neurological outcome in in-hospital (IHCA) or out-of-hospital (OHCA) cardiac arrest (Suppl Table 7); a multivariable regression analysis to identify independent predictors of lymphopenia on admission in patients without therapy with corticosteroids or other immunosoppressive drugs (Suppl Table 8).

## Abbreviations

CA: cardiac arrest
CPR: cardiopulmonary resuscitation
CPC: cerebral performance category score
CRRT: continuous renal replacement therapy
ECMO: extracorporeal membrane oxygenation
IABP: intra-aortic balloon counterpulsation
ICU: intensive care unit
IHCA: in-hospital cardiac arrest
OHCA: out-of-hospital cardiac arrest
PCAS: post-cardiac arrest syndrome
ROSC: return of spontaneous circulation
TTM: targeted temperature management
