# Supplementary material for: Lymphopaenia in cardiac arrest patients
Source: Ann Intensive Care. 2017 Aug 14;7:85. doi: 10.1186/s13613-017-0308-z (PMC5555958; doi:10.1186/s13613-017-0308-z)
Supplement: Supplementary file 1 — Additional file 1. This documents includes comparisons between patients with in-hospital (IHCA) and out-of-hospital cardiac arrest (OHCA—Suppl Table 1); analysis of mortality and neurological outcome in IHCA (Suppl Table 2) and OHCA (Suppl Table 3) patients; the characteristics of lymphopenic patients after having excluded those receiving immunosuppressive therapies (Suppl Table 4); a multivariable regression analysis to identify independent predictors of lymphopenia on admission in in-hospital (IHCA) or out-of-hospital (OHCA) cardiac arrest (Suppl Table 5); a multivariable regression analysis to identify independent predictors of ICU outcome in in-hospital (IHCA) or out-of-hospital (OHCA) cardiac arrest (Suppl Table 6); a multivariable regression analysis to identify independent predictors of long-term neurological outcome in in-hospital (IHCA) or out-of-hospital (OHCA) cardiac arrest (Suppl Table 7); a multivariable regression analysis to identify independent predictors of lymphopenia on admission in patients without therapy with corticosteroids or other immunosoppressive drugs (Suppl Table 8). [file 13613_2017_308_MOESM1_ESM.docx]

***Supplemental Material***

**Lymphopaenia in cardiac arrest patients**

Paola Villois^1^, David Grimaldi^1^, Savino Spadaro^2^, Claudia Righy Shinotsuka^1^,

Vito Fontana^1^, Sabino Scolletta^3^, Federico Franchi^3^, Jean-Louis Vincent^1^,

Jacques Creteur^1^, Fabio Silvio Taccone^1^

*^1^Department of Intensive Care*

*Erasme Hospital*

*Université Libre de Bruxelles*

*Route de Lennik, 808*

*1070 Brussels, Belgium*

[*paolavillois@gmail.com*](mailto:paolavillois@gmail.com)

[*david.grimaldi@erasme.ulb.ac.be*](mailto:david.grimaldi@erasme.ulb.ac.be)

[*claudiarighy@hotmail.com*](mailto:claudiarighy@hotmail.com)

[*fontanapv@hotmail.it*](mailto:fontanapv@hotmail.it)

[*jlvincen@intensive.org*](mailto:jlvincen@intensive.org)

[*jcreteur@ulb.ac.be*](mailto:jcreteur@ulb.ac.be)

[*ftaccone@ulb.ac.be*](mailto:ftaccone@ulb.ac.be)

*^2^Department of Morphological Surgery and Experimental Medicine*

*Arcispedale Sant’Anna*

*Università di Ferrara*

*Via AldoMoro,8*

*44121 Ferrara, Italy*

[*savinospadaro@gmai.com*](mailto:savinospadaro@gmai.com)

*^3^Department of Anesthesia and Intensive Care*

*Policlinico Santa Maria alle Scotte*

*Universitá di Siena*

*Viale Bracci, 14*

*53100 Siena, Italy*

[*sabino.scolletta@dbm.unisi.it*](mailto:sabino.scolletta@dbm.unisi.it)

[*federico.franchi@dbm.unisi.it*](mailto:federico.franchi@dbm.unisi.it)

Corresponding author:

Pr. **Fabio Silvio TACCONE**

Department of Intensive Care

Erasme Hospital

Université Libre de Bruxelles (ULB)

Route de Lennik, 808

1070 – Brussels (BELGIUM)

email: ftaccone@ulb.ac.be

tel: +322 555 5587

fax: +322 555 4698

**Supplemental Table 1.** Characteristics of study population according to the presence of lymphopenia on admission in patients with in-hospital or out-of-hospital cardiac arrest.

|  | **In-Hospital Cardiac Arrest** | | **Out-of-Hospital Cardiac Arrest** | |
| --- | --- | --- | --- | --- |
|  | **Lymphopenia**  **(n=91)** | **No Lymphopenia**  **(n=72)** | **Lymphopenia**  **(n=60)** | **No Lymphopenia**  **(n=151)** |
| Age, years | 71 [58-80] | 72 [63-72]^*^ | 64 [51-76] | 58 [50-71]^#^ |
| Weight, kgs | 75 [67-85] | 76 [65-89] | 75 [68-80] | 77 [67-86]^#^ |
| Male gender, n (%) | 63 (69) | 48 (67) | 42 (70) | 108 (71) |
|  |  |  |  |  |
| Witnessed CA, n (%) | 86 (94) | 70 (97) | 46 (77) | 114 (75) |
| Bystander CPR, n (%) | 83 (91) | 62 (86) | 27 (45) | 76 (50) |
| Time to ROSC, min | 10 [5-18] | 13 [7-21]^#^ | 15 [7-25]^*^ | 20 [13-30] |
| Epinephrine, mg | 2 [1-4] | 3 [1-5]^#^ | 3 [2-5] | 4 [2-7]^#^ |
| Cardiac origin of arrest, n (%) | 46 (51) | 35 (49) | 34 (57) | 111 (74)^*^ |
| Shockable rhythm, n (%) | 19 (22) | 23 (32) | 21 (35) | 81 (54)^*^ |
|  |  |  |  |  |
| *COMORBIDITIES* |  |  |  |  |
| Chronic heart failure, n (%) | 27 (30) | 23 (32) | 10 (17) | 21 (14) |
| Hypertension, n (%) | 53 (58) | 32 (44) | 25 (42) | 59 (39) |
| Coronary artery disease, n (%) | 33 (36) | 31 (43) | 29 (48) | 70 (46) |
| Diabetes, n (%) | 35 (38) | 20 (28) | 13 (22) | 22 (15) |
| COPD/asthma, n (%) | 19 (21) | 11 (15) | 12 (20) | 22 (15) |
| Neurological disease, n (%) | 15 (16) | 15 (21) | 17 (28) | 17 (11)^*^ |
| Liver cirrhosis, n (%) | 11 (12) | 5 (7) | 3 (5) | 3 (2) |
| HIV, n (%) | 1 (1) | 0 (0) | 0 (0) | (0) |
| Corticosteroid therapy, n (%) | 28 (31) | 10 (14)^*^ | 13 (22) | 20 (13)^#^ |
| Other immunosuppressive agents, n (%) | 8 (9) | 2 (3) | 3 (5) | 2 (1)^#^ |
|  |  |  |  |  |
| *INITIAL BLOOD ANALYSIS* |  |  |  |  |
| White Blood Cells, /mm^3^ | 10500 [9000-16700] | 12600 [9475-19000]^*^ | 10950 [7825-14575] ^*^ | 13400 [10100-17200] |
| Hemoglobin, g/dL | 12.0 [9.9-13.7] | 10.6 [8.9-12.9]^#^ | 12.1 [10.3-14.0] ^*^ | 13.3 [12.2-14.3] |
| Platelets, *10^3^/mm^3^ | 149 [89-241] | 211 [122-329]^*^ | 160 [128-226] ^*^ | 209 [167-276] |
| C-reactive protein, mg/dL | 50 [13-140] | 21 [6-88]^*^ | 6.5 [1.3-43.5] ^*^ | 2.9 [1.1-9.2] |
|  |  |  |  |  |
| *DURING ICU STAY* |  |  |  |  |
| Lactate on admission, mEq/L | 3.4 [2.2-6.1] | 4.5 [2.7-6.8] | 4.7 [3.1-9.5] | 4.4 [3-8.5] |
| Infection, n (%) | 67 (74) | 45 (62) | 33 (55) | 67 (44)^#^ |
| IABP, n (%) | 3 (3) | 8 (11) | 3 (5) | 12 (8) |
| ECMO, n (%) | 10 (11) | 9 (12) | 13 (22) | 19 (13)^#^ |
| Shock, n (%) | 54 (59) | 50 (69) | 36 (60) | 63 (42)^*^ |
| Acute kidney Injury, n (%) | 31 (35) | 19 (26) | 5 (8) | 12 (8) |
| CRRT, n (%) | 25 (27) | 13 (18) | 5 (8) | 13 (9) |
| Vasopressor therapy, n (%) | 71 (78) | 60 (83) | 49 (82) | 91 (60)^*^ |
| Dobutamine therapy, n (%) | 53 (58) | 42 (58) | 32 (53) | 71 (47) |
|  |  |  |  |  |
| *OUTCOMES* |  |  |  |  |
| ICU mortality, n (%) | 53 (58) | 37 (51) | 42 (70)^*^ | 83 (55) |
| UO at 3 months, n (%) | 60 (66) | 46 (64) | 48 (80) | 89 (59)^*^ |

CA = cardiac arrest; CPR = cardiopulmonary resuscitation; ROSC = return of spontaneous circulation; COPD = chronic obstructive pulmonary disease; IABP = intra-aortic balloon pump counterpulsation; ECMO = extracorporeal membrane oxygenation; CRRT = continuous renal replacement therapy; ICU = intensive care unit; UO = Unfavorable neurological outcome.

^*^ = p<0.05; ^#^ = p<0.2

**Supplemental Table 2.** Characteristics of in-hospital cardiac arrest patients according to the ICU and long-term neurological outcome.

|  | **ICU Survivors**  **(n=73)** | **ICU Non-Survivors**  **(n=90)** | **FO**  **(n=57)** | **UO**  **(n=106)** |
| --- | --- | --- | --- | --- |
| Age, years | 66 [52-75] | 68 [55-81]^#^ | 64 [52-73] | 68 [55-79]^*^ |
| Weight, kgs | 77 [68-90] | 75 [65-85] | 79 [73-90] | 75 [65-85] |
| Male gender, n (%) | 50 (68) | 61 (70) | 40 (70) | 71 (67) |
|  |  |  |  |  |
| Witnessed CA, n (%) | 72 (99) | 84 (93)^#^ | 56 (98) | 100 (94) |
| Bystander CPR, n (%) | 69 (94) | 76 (84)^*^ | 55 (96) | 90 (85)^*^ |
| Time to ROSC, min | 8 [4-15] | 15 [7-20]^*^ | 8 [4-15] | 13 [6-20] |
| Epinephrine, mg | 2 [1-4] | 3 [2-5]^*^ | 2 [1-4] | 3 [2-5]^*^ |
| Cardiac origin of arrest, n (%) | 21 (41) | 60 (59)^*^ | 35 (61) | 46 (43)^*^ |
| Shockable rhytm, n (%) | 27 (37) | 16 (18)^*^ | 24 (42) | 19 (18)^*^ |
|  |  |  |  |  |
| *INITIAL BLOOD ANALYSIS* |  |  |  |  |
| White Blood Cells, /mm^3^ | 11800 [8100-15300] | 12650 [8500-17500] | 11600 [8100-15100] | 12050 [8475-17850] |
| Hemoglobin, g/dL | 11.0 [9.0-13.0] | 10.0 [8.09-12.0]^*^ | 11.01 [9.0-14.0] ^*^ | 10.0 [9.0-12.0] |
| Platelets, *10^3^/mm^3^ | 174 [122-263] | 150 [83-280]^#^ | 188 [124-268] ^*^ | 149 [87-269] |
| C-reactive protein, mg/dL | 31 [6-80] | 46 [12-152]^*^ | 27.0 [3.6-74.0] ^*^ | 49.5 [14.7-140.0] |
|  |  |  |  |  |
| *COMORBIDITIES* |  |  |  |  |
| Chronic heart failure, n (%) | 22 (30) | 28 (31) | 17 (30) | 33 (31) |
| Hypertension, n (%) | 39 (53) | 46 (51) | 31 (54) | 54 (51) |
| Coronary artery disease, n (%) | 30 (41 | 34 (39) | 22 (39) | 42 (40) |
| Diabetes, n (%) | 21 (29) | 34 (38) | 15 (26) | 40 (38)^#^ |
| COPD/asthma, n (%) | 17 (23) | 13 (14)^#^ | 12 (21) | 18 (17) |
| Neurological disease, n (%) | 14 (19) | 16 (18) | 8 (14) | 22 (21) |
| Liver cirrhosis, n (%) | 5 (7) | 11 (12) | 3 (5) | 13 (12)^#^ |
| HIV, n (%) | 1 (1) | 0 (0) | 1 (2) | 0 (0) |
| Corticosteroid therapy, n (%) | 14 (19) | 24 (27) | 11 (19) | 27 (25) |
| Immunosuppressive agents, n (%) | 4 (5) | 6 (7) | 2 (3) | 8 (7) |
|  |  |  |  |  |
| *DURING ICU STAY* |  |  |  |  |
| Lactate on admission, mEq/L | 3.3 [2.0-5.3] | 4.4 [2.7-7.8]^*^ | 3.8 [2.0-5.4] | 4.1 [2.6-7.2]^*^ |
| Infection, n (%) | 43 (59) | 69 (77)^*^ | 32 (56) | 80 (75)^*^ |
| IABP, n (%) | 5 (7) | 6 (7) | 3 (5) | 8 (7) |
| ECMO, n (%) | 9 (12) | 10 (11) | 9 (16) | 10 (9) |
| Shock, n (%) | 38 (52) | 66 (73)^*^ | 31 (54) | 73 (69)^#^ |
| Acute kidney Injury, n (%) | 22 (30) | 28 (32) | 16 (28) | 34 (33) |
| CRRT, n (%) | 16 (22) | 22 (24) | 13 (23) | 25 (24) |
| Vasopressor therapy, n (%) | 54 (74) | 77 (86)^#^ | 43 (75) | 88 (83)^#^ |
| Dobutamine therapy, n (%) | 40 (55) | 55 (61) | 32 (56) | 63 (59) |
| Lymphopenia. n (%) | 38 (52) | 53 (59) | 31 (54) | 60 (57) |

CA = cardiac arrest; CPR = cardiopulmonary resuscitation; ROSC = return of spontaneous circulation; COPD = chronic obstructive pulmonary disease; IABP = intra-aortic balloon pump counterpulsation; ECMO = extracorporeal membrane oxygenation; CRRT = continuous renal replacement therapy; ICU = intensive care unit.

* p < 0.05, **^#^** p<0.2 in survivors vs. non-survivors OR favorable outcome (FO) vs. unfavorable outcome (UO)

**Supplemental Table 3.** Characteristics of out-of-hospital cardiac arrest patients according to the ICU and long-term neurological outcome.

|  | **ICU Survivors**  **(n=86)** | **ICU Non-Survivors**  **(n=125)** | **FO**  **(n=74)** | **UO**  **(n=137)** |
| --- | --- | --- | --- | --- |
| Age, years | 54 [48-62] | 64 [53-77]^*^ | 54 [49-63] | 63 [52-76]^*^ |
| Weight, kgs | 77 [70-82] | 75 [65-85] | 77 [70-83] | 75 [65-85] |
| Male gender, n (%) | 67 (78) | 83 (64)^#^ | 57 (77) | 93 (68)^#^ |
|  |  |  |  |  |
| Witnessed CA, n (%) | 71 (83) | 89 (71)^#^ | 62 (83) | 98 (71)^*^ |
| Bystander CPR, n (%) | 52 (60) | 51 (41)^*^ | 46 (62) | 57 (42)^*^ |
| Time to ROSC, min | 15 [10-25] | 21 [13-30]^*^ | 15 [9-25] | 20 [13-30]^*^ |
| Epinephrine, mg | 3 [2-5] | 5 [2-8] ^*^ | 3 [2-5] | 5 [2-5]^*^ |
| Cardiac origin of arrest, n (%) | 67 (78) | 78 (62)^*^ | 59 (80) | 86 (63)^*^ |
| Shockable rhytm, n (%) | 62 (72) | 40 (32)^*^ | 58 (78) | 44 (32)^*^ |
|  |  |  |  |  |
| *INITIAL BLOOD ANALYSIS* |  |  |  |  |
| White Blood Cells, /mm^3^ | 12450 [9775-16800] | 12300 [9000-16750] | 136000 [10375-16925] | 11900 [8800-16450] |
| Hemoglobin, g/dL | 14.0 [12.0-15.0] | 13.0 [11.0-14.0]^*^ | 14.0 [12.0-15.0] | 13.0 [11.0-14.0]^*^ |
| Platelets, *10^3^/mm^3^ | 208 [158-267] | 201 [137-245] | 207 [158-269] | 201 [141-242]^#^ |
| C-reactive protein, mg/dL | 2.1 [0.8-8.1] | 4.0 [1.3-25.0]^#^ | 2.0 [1.0-7.4] | 4.0 [1.3-27.5]^*^ |
|  |  |  |  |  |
| *COMORBIDITIES* |  |  |  |  |
| Chronic heart failure, n (%) | 11 (13) | 20 (16) | 10 (13) | 21 (15) |
| Hypertension, n (%) | 37 (43) | 47 (38) | 29 (39) | 55 (40) |
| Coronary artery disease, n (%) | 37 (43) | 62 (50) | 32 (43) | 67 (49) |
| Diabetes, n (%) | 13 (15) | 22 (10) | 10 (13) | 25 (18) |
| COPD/asthma, n (%) | 8 (9) | 26 (21)^*^ | 7 (9) | 27 (20)^#^ |
| Neurological disease, n (%) | 8 (9) | 26 (21)^*^ | 5 (7) | 29 (21)^*^ |
| Liver cirrhosis, n (%) | 2 (2) | 4 (3) | 1 (1) | 5 (4) |
| HIV, n (%) | 0 (0) | 0 (0) | 0 (0) | 0 (0) |
| Corticosteroid therapy, n (%) | 10 (12) | 23 (18) | 9 (12) | 24 (17) |
| Immunosuppressive agents, n (%) | 1 (1) | 4 (3) | 1 (1) | 4 (3) |
|  |  |  |  |  |
| *DURING ICU STAY* |  |  |  |  |
| Lactate on admission, mEq/L | 4.0 [2.8-6.2] | 5.1 [2.5-8.0]^*^ | 4.1 [2.8-6.6] | 4.9 [3.3-9.4]^*^ |
| Infection, n (%) | 61 (71) | 39 (31)^*^ | 51 (69) | 49 (36)^*^ |
| IABP, n (%) | 4 (5) | 11 (9) | 4 (5) | 11 (8) |
| ECMO, n (%) | 10 (12) | 22 (18) | 9 (12) | 23 (17) |
| Shock, n (%) | 29 (34) | 70 (56)^*^ | 27 (36) | 72 (53)^*^ |
| Acute kidney Injury, n (%) | 4 (5) | 13 (10)^#^ | 4 (5) | 13 (9) |
| CRRT, n (%) | 7 (8) | 11 (9) | 6 (8) | 12 (9) |
| Vasopressor therapy, n (%) | 44 (51) | 96 (77)^*^ | 38 (51) | 102 (74)^*^ |
| Dobutamine therapy, n (%) | 40 (46) | 63 (50) | 34 (46) | 69 (50) |
| Lymphopenia. n (%) | 18 (21) | 42 (34)^#^ | 12 (16) | 48 (35)^*^ |

CA = cardiac arrest; CPR = cardiopulmonary resuscitation; ROSC = return of spontaneous circulation; COPD = chronic obstructive pulmonary disease; IABP = intra-aortic balloon pump counterpulsation; ECMO = extracorporeal membrane oxygenation; CRRT = continuous renal replacement therapy; ICU = intensive care unit.

* p < 0.05, **^#^** p<0.2 in survivors vs. non-survivors OR favorable outcome (FO) vs. unfavorable outcome (UO)

**Supplemental Table 4.** Characteristics of study population according to the presence of lymphopenia on admission in patients without corticosteroid or immunosuppressive therapies.

|  | **No Lymphopenia**  **(n=194)** | **Lymphopenia**  **(n=107)** | **p value** |
| --- | --- | --- | --- |
| Age, years | 58 [50-73] | 71 [59-79] | <0.001 |
| Weight, kgs | 77 [66-85] | 75 [65-80] | 0.454 |
| Male gender, n (%) | 137 (71) | 76 (71) | 0.940 |
|  |  |  |  |
| Witnessed CA, n (%) | 157 (81) | 90 (84) | 0.490 |
| Bystander CPR, n (%) | 123 (63) | 75 (70) | 0.242 |
| Time to ROSC, min | 16 [10-25] | 10 [5-20] | <0.001 |
| Epinephrine, mg | 4 [2-7] | 3 [1-4] | 0.001 |
| Cardiac origin of arrest, n (%) | 131 (67) | 62 (58) | 0.097 |
| Shockable rhythm, n (%) | 94 (48) | 27 (25) | <0.001 |
| Out of Hospital CA, n (%) | 131 (68) | 46 (43) | <0.001 |
|  |  |  |  |
| *COMORBIDITIES* |  |  |  |
| Chronic heart failure, n (%) | 38 (20) | 25 (23) | 0.441 |
| Hypertension, n (%) | 80 (41) | 59 (55) | 0.021 |
| Coronary artery disease, n (%) | 90 (46) | 48 (45) | 0.798 |
| Diabetes, n (%) | 32 (16) | 33 (31) | 0.004 |
| COPD/asthma, n (%) | 26 (13) | 22 (21) | 0.144 |
| Neurological disease, n (%) | 29 (15) | 25 (23) | 0.069 |
| Liver cirrhosis, n (%) | 8 (4) | 8 (7) | 0.283 |
| HIV, n (%) | 0 (0) | 1 (1) | 0.355 |
|  |  |  |  |
| *INITIAL BLOOD ANALYSIS* |  |  |  |
| White Blood Cells, /mm^3^ | 13100 [10025-17825] | 10700 [7900-4200] | <0.001 |
| Hemoglobin, g/dL | 13.0 [11.0-14.0] | 11 [9-14] | <0.001 |
| Platelets, *10^3^/mm^3^ | 214 [158-289] | 169 [121-169] | 0.005 |
| C-reactive protein, mg/dL | 4.4 [1.5-21.0] | 28 [2.5-69] | <0.001 |
|  |  |  |  |
| *DURING ICU STAY* |  |  |  |
| Lactate on admission, mEq/L | 4.3 [2.9-7.2] | 3.9 [2.5-6-7] | 0.471 |
| Infection, n (%) | 101 (52) | 63 (59) | 0.256 |
| IABP, n (%) | 18 (9) | 4 (4) | 0.104 |
| ECMO, n (%) | 19 (10) | 10 (9) | 1.000 |
| Shock, n (%) | 89 (46) | 55 (51) | 0.358 |
| Acute kidney Injury, n (%) | 25 (13) | 20 (19) | 0.178 |
| CRRT, n (%) | 20 (10) | 19 (18) | 0.074 |
| Vasopressor therapy, n (%) | 126 (65) | 80 (75) | 0.079 |
| Dobutamine therapy, n (%) | 95 (49) | 56 (52) | 0.576 |
|  |  |  |  |
| *OUTCOMES* |  |  |  |
| ICU mortality, n (%) | 99 (51) | 68 (64) | 0.036 |
| UO at 3 months, n (%) | 113 (58) | 78 (73) | 0.012 |

CA = cardiac arrest; CPR = cardiopulmonary resuscitation; ROSC = return of spontaneous circulation; COPD = chronic obstructive pulmonary disease; IABP = intra-aortic balloon pump counterpulsation; ECMO = extracorporeal membrane oxygenation; CRRT = continuous renal replacement therapy; ICU = intensive care unit; UO = Unfavorable neurological outcome.

**Supplemental Table 5.** Multivariable regression analysis to identify independent predictors of lymphopenia on admission in in-hospital (IHCA) or out-of-hospital (OHCA) cardiac arrest.

|  | **IHCA** | | | |
| --- | --- | --- | --- | --- |
|  | **p value** | **OR** | **95% CI for OR** | |
|  |  |  | **Lower** | **Upper** |
| **Age, years** | 0.004 | 1.036 | 1.012 | 1.061 |
| **Time to ROSC** | 0.02 | 0.959 | 0.925 | 0.994 |
| **White blood cells, 10^3^/mm^3^** | 0.025 | 1.056 | 1.023 | 1.067 |
| **C-reactive protein, mg/dL** | 0.04 | 1.005 | 1.001 | 1.010 |
| **Corticosteroids** | 0.005 | 3.971 | 1.529 | 10.311 |
|  |  |  |  |  |
|  | **OHCA** | | | |
|  | **p value** | **OR** | **95% CI for OR** | |
|  |  |  | **Lower** | **Upper** |
| **Time to ROSC** | 0.003 | 0.956 | 0.928 | 0.985 |
| **White blood cells, 10^3^/mm^3^** | 0.009 | 1.078 | 1.023 | 1.143 |
| **C-reactive protein, mg/dL** | 0.002 | 1.011 | 1.004 | 1.018 |
| **Platelets, 10^3^/mm^3^** | 0.04 | 0.995 | 0.991 | 0.999 |

ROSC = return of spontaneous circulation

**Supplemental Table 6.** Multivariable regression analysis to identify independent predictors of ICU outcome in in-hospital (IHCA) or out-of-hospital (OHCA) cardiac arrest.

|  | **IHCA – ICU Mortality** | | | |
| --- | --- | --- | --- | --- |
|  | **p value** | **OR** | **95% CI for OR** | |
|  |  |  | **Lower** | **Upper** |
| **Bystander CPR** | 0.04 | 0.253 | 0.061 | 0.931 |
| **Non-shockable rhythm** | 0.04 | 2.311 | 1.046 | 5.104 |
| **Epinephrine dose, mg** | 0.048 | 1.128 | 1.002 | 1.276 |
| **Shock** | 0.03 | 2.187 | 1.076 | 4.446 |
|  |  |  |  |  |
|  | **OHCA – ICU Mortality** | | | |
|  | **p value** | **OR** | **95% CI for OR** | |
|  |  |  | **Lower** | **Upper** |
| **Bystander CPR** | 0.049 | 1.068 | 1.009 | 1.096 |
| **Non-cardiac origin** | 0.005 | 3.612 | 1.475 | 8.847 |
| **Non-shockable rhythm** | 0.003 | 3.043 | 1.453 | 6.373 |
| **Age, years** | 0.01 | 1.083 | 1.056 | 1.125 |
| **Lactate on Admission, mmol/L** | 0.005 | 1.151 | 1.043 | 1.270 |

CPR = cardiopulmonary resuscitation

**Supplemental Table 7.** Multivariable regression analysis to identify independent predictors of long-term neurological outcome in in-hospital (IHCA) or out-of-hospital (OHCA) cardiac arrest.

|  | **IHCA – UO** | | | |
| --- | --- | --- | --- | --- |
|  | **p value** | **OR** | **95% CI for OR** | |
|  |  |  | **Lower** | **Upper** |
| **Bystander CPR** | 0.046 | 0.207 | 0.044 | 0.971 |
| **Non-shockable rhythm** | 0.001 | 3.318 | 1.584 | 6.949 |
|  |  |  |  |  |
|  | **OHCA – UO** | | | |
|  | **p value** | **OR** | **95% CI for OR** | |
|  |  |  | **Lower** | **Upper** |
| **Age, years** | 0.001 | 1.044 | 1.018 | 1.070 |
| **Non-shockable rhythm** | <0.001 | 6.675 | 3.273 | 13.610 |
| **Lymphopenia** | 0.016 | 2.754 | 1.206 | 6.288 |
| **Epinephrine, mg** | 0.001 | 1.200 | 1.077 | 1.337 |

CPR = cardiopulmonary resuscitation; UO = unfavourable neurological outcome

**Supplemental Table 8.** Multivariable regression analysis to identify independent predictors of lymphopenia on admission in patients without therapy with corticosteroids or other immunosoppressive drugs (n=301).

|  | **Lymphopenia** | | | |
| --- | --- | --- | --- | --- |
|  | **p value** | **OR** | **95% CI for OR** | |
|  |  |  | **Lower** | **Upper** |
| **Age, years** | 0.009 | 1.024 | 1.006 | 1.043 |
| **Time to ROSC, min** | 0.001 | 0.957 | 0.932 | 0.982 |
| **White Blood Cells, 10^3^/mm^3^** | 0.001 | 1.034 | 1.018 | 1.097 |
| **CRP, mg/dL** | 0.005 | 1.006 | 1.002 | 1.011 |
| **Platelets, 10^3^/mm^3^** | 0.012 | 0.996 | 0.994 | 0.999 |
| **Non-shockable rhythm** | 0.019 | 2.024 | 1.123 | 3.646 |

ROSC = return of spontaneous circulation; CRP = C-reactive protein
